# CO Oxidation over Pd Catalyst Supported on Porous TiO_2_ Prepared by Plasma Electrolytic Oxidation (PEO) of a Ti Metallic Carrier

**DOI:** 10.3390/ma15124301

**Published:** 2022-06-17

**Authors:** Payam Samadi, Michal J. Binczarski, Aleksandra Pawlaczyk, Jacek Rogowski, Malgorzata I. Szynkowska-Jozwik, Izabela A. Witonska

**Affiliations:** Institute of General and Ecological Chemistry, Lodz University of Technology, 116 Zeromskiego Street, 90-924 Lodz, Poland; payam.samadi@edu.p.lodz.pl (P.S.); michal.binczarski@p.lodz.pl (M.J.B.); aleksandra.pawlaczyk@p.lodz.pl (A.P.); jacek.rogowski@p.lodz.pl (J.R.); malgorzata.szynkowska-jozwik@p.lodz.pl (M.I.S.-J.)

**Keywords:** PEO, CO oxidation, preparation of porous layer, preparation of Pd catalyst by adsorption, XRD, ICP, ToF-SIMS

## Abstract

A porous TiO_2_ layer was prepared with the plasma electrolytic oxidation (PEO) of Ti. In a further step, Pd was deposited on the TiO_2_ surface layer using the adsorption method. The activity of the Pd/TiO_2_/Ti catalyst was investigated during the oxidation of CO to CO_2_ in a mixture of air with 5% CO. The structure of the catalytic active layer was studied using a scanning electron microscope equipped with an energy dispersive spectrometer (SEM-EDS), time-of-flight secondary ion mass spectrometry (TOF-SIMS), inductively coupled plasma mass spectrometry (ICP-MS), and X-ray diffraction (XRD). The PEO process provided a porous TiO_2_ layer with a uniform thickness in the range of 5–10 µm, which is desirable for the production of Pd-supported catalysts. A TOF-SIMS analysis showed the formation of Pd nanoparticles after the adsorption treatment. The conversion of CO to CO_2_ in all samples was achieved at 150–280 °C, depending on the concentration of Pd. The composition of Pd/ TiO_2_/Ti was determined using ICP-MS. The optimum concentration of Pd on the surface of the catalyst was approximately 0.14% wt. This concentration was obtained when a 0.4% PdCl_2_ solution was used in the adsorption process. Increasing the concentration of PdCl_2_ did not lead to a further improvement in the activity of Pd/ TiO_2_/Ti.

## 1. Introduction

Air pollution from various sources poses a severe danger to human health and the environment globally [1,2]. Carbon monoxide is one of the most toxic exhaust pollutants. In many regions around the world, especially highly urbanized areas, the concentration of carbon monoxide is far higher than permitted by air quality standards [3]. The conversion of carbon monoxide into carbon dioxide by photocatalytic adsorption, physicochemical adsorption, or electrocatalysis has long been regarded as one of the most efficient methods of dealing with this issue [4,5,6,7]. Different types of catalysts have been investigated for this purpose. Metallic support catalysts with properties such as thermal stability, corrosion resistance, and the capacity to be shaped have attracted much attention [8]. An adequate pretreatment not only improves the adhesion of catalytic coatings, but also extends the life of the metallic support catalyst [9,10].

Various techniques can be used to prepare metallic substrates for the application of a functional oxide layer. One technique for treating titanium material is traditional anodizing, whereby the surface is treated anodically to produce a porous oxide layer. Another technique is plasma electrolytic oxidation (PEO) [11,12,13,14,15]. PEO is an anodic electrochemical treatment that results in uniform sparks on the surface. An adhesive porous layer of oxides forms on the surface, which facilitates the preparation of metallic catalysts [16,17]. PEO has several advantages over traditional anodizing, such as providing a better corrosion resistivity and hardness [18]. Thicker TiO_2_ coatings and stronger adhesion between the coating and substrate can also be achieved using PEO. This is very important for applications of the structure as a catalyst support. From an industrial point of view, PEO is preferable because it does not require a special pretreatment of the metallic material [19,20].

Commercial catalysts for CO oxidation are typically non-noble metal oxide catalysts, such as the mesoporous Cu–Mn Hopcalite catalyst [21], or mixtures of CuO, Ce_3_O_4_, and NiO with CeO_2_, ZnO, TiO_2_ [22,23,24], SiO_2_ [25], or Al_2_O_3_ [26,27]. An alternative solution is to use noble-metal-supported catalysts, such as Pt, Au, Pd, or Ru [28,29,30,31]. One of the most widely investigated catalysts is Pd supported on various metal oxides. The CO oxidation ability of a Pd-supported catalyst depends strongly on the nature of the support [32,33,34]. For example, a 50% conversion of CO (T_50_) occurs as follows: CeO_2_ (45 °C) < ZrO_2_ (165 °C) < TiO_2_ (185 °C) < Al_2_O_3_ (205 °C) < SiO_2_ (255 °C) [35]. In general, the catalytic activity of noble-metal-supported systems is connected with the expansion of the carrier surface. For example, the use of nanomaterials such as nanotubes or nanowires as supports for metallic catalysts may increase the dispersion of the active metal, as well as the stability and selectivity of the system [36]. As a result of electron interactions between the active metal and the nano-structured support, the crystallite size of the noble metal is significantly reduced and the activity of the catalyst is increased [37]. In the case of the oxidation of CO, the temperature at which a 100% conversion of CO is achieved drops significantly [38,39].

Nanostructured TiO_2_ has been investigated as a possible support for metallic catalysts [40,41,42]. Kokuvecz et al. [40] presented methods for the preparation of TiO_2_ nanowires and nanotubes. The physicochemical characteristics were also discussed, as well as the possibility of modifying their surfaces with covalent functionalization, ion exchange, and metal loading. Despite the interesting properties of nanostructured powdered materials, their use as carriers in metallic CO catalysts is hindered by the high resistance of the flowing gases. From the industrial point of view, it is preferable to produce porous structures on the surfaces of construction materials (e.g., Ti used for the preparation of a monolithic structure), as effective parameters related to the pressure drop, such as the channel size, can be designed more freely [43].

The PEO technique has been investigated for the preparation of a porous structure over construction materials such as Ti, Al, and Mg [44,45,46,47]. Both noble and non-noble metals have been investigated for their CO oxidation activity on supports prepared with PEO [48,49,50]. Different techniques have been applied to deposit these metals on the supports, including adding the elements to the PEO electrolyte or post-treatments after PEO such as wet impregnation, deposition precipitation, or sol-gel [35,44,51].

The main goal of this study was to prepare a Pd catalyst supported on TiO_2_/Ti using the adsorption method. To the best of the authors’ knowledge, there have been no previous studies in which metal-supported catalysts were prepared using a combination of PEO and the adsorption method. SEM-EDS, TOF-SIMS, ICP-MS, and XRD were used to characterize the structure and composition of the Pd/TiO_2_/Ti catalysts. The catalytic activity of the samples was tested using the CO oxidation process.

## 2. Materials and Methods

### 2.1. Materials

Samples were composed of commercial Ti (Baoji Taihengte Metal CO., Ltd., Baoji, China) grade II (99.3% Ti, 0.3% Fe, 0.1% C) in two different shapes. Flat samples 0.1 mm in thickness were used to determine the elemental and phase composition of the surface. Samples in the shape of a wire (0.6 mm in diameter with a working surface area of 3 cm^2^) were used to test the catalytic activity. The solution used for the PEO process was based on sulfuric acid (H_2_SO_4_, 85%, Chempur, Piekary Slaskie, Poland). The samples were placed in the solution without surface pretreatment.

### 2.2. Preparation of Porous Layers of TiO_2_ on Ti Materials with PEO

**Figure 1:**
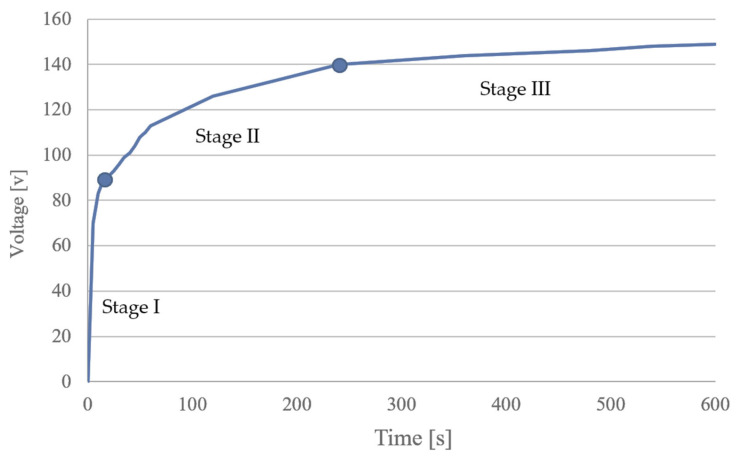
Voltage–time plot during plasma electrolytic oxidation (PEO).

The support of the catalyst was prepared with PEO. The set-up included a high-power voltage source (EA-PSI model 8360-15 T, Elektro-Automatic GmbH & CO.KG, Viersen, Germany), electrical connections, an electrochemical cell (with the Ti sample as the anode and a carbon rod as the cathode), a mixer (Model RZR 2021, Heidolph Instruments GmbH & Co. KG, Schwabach, Germany), a temperature fixing bath (Model GD100, Grant Instruments Ltd., Cambridge, UK), and an exhaust hood. The current density and voltage were 0.2 A/cm^2^ and 150 V, respectively. During the process, the voltage–time plot was as shown in Figure 1. The plot followed the main pattern of the PEO process, which had three phases corresponding to anodic oxidation (Stage I), breakdown voltage (Stage II), and steady state (Stage III). After PEO treatment, the samples of TiO_2_/Ti materials were dried until reaching a constant weight at 110 °C using a moisture analyzer (MA 50.R Moisture Analyzer, model MA 50.R, RADWAG, Radom, Poland) and subjected to microscopic and XRD examinations.

### 2.3. Preparation of Pd/TiO_2_/Ti Catalysts by the Adsorption Method

The TiO_2_/Ti materials obtained with PEO were placed into a solution of Pd (PdCl_2_, Avantor, Gliwice, Poland) acidified with HCl to pH ~4 (HCl, Avantor, Gliwice, Poland). The solution contained between 0.02% and 2.0% palladium. Previously, we investigated the optimal timing for the adsorption (from 1 min to 120 h). The optimum time of adsorption was 5 min at room temperature. Therefore, TiO_2_/Ti materials were kept in the solution for 5 min at room temperature. The samples were removed from the Pd solution, washed with the solvent, and dried using a moisture analyzer (MA 50.R Moisture Analyzer, model MA 50.R, RADWAG, Radom, Poland) at 110 °C to a constant weight, followed by reduction in H_2_ at 400 °C for approximately 2 h.

### 2.4. Catalytic Activity Test

The catalytic activity of the samples was tested in the CO oxidation reaction using a tube-shaped quartz reactor with mass flow controller. The reactor was equipped with a digital heating system and temperature controller. A gas chromatography (GC) analyzer was also used, equipped with a thermal conductivity detector (TCD) and packed column (Restek packed column, ShinCarbon ST 80/100, 2 m × 2 mm lD × 1/8′′ OD Silco HP, Bellefonte, PA, USA), with helium as a carrier gas. The temperature of the GC was maintained at 50 °C. The retention times for CO and CO_2_ were approximately 2 min and 18 min, respectively. Three wire samples were attached together and placed in the active zone of the quartz tube reactor. The active zone was 1 cm in diameter and 3 cm in height. The weight of the samples was approximately 0.5 g in each test. The composition of inlet gas was 5% CO and 95% air (Linde Gaz Polska, Krakow, Poland). The gas flow rate was 50 mL/min. Every 20 min, 500 µL of the gas was injected into the GC analyzer and the outlet concentrations of CO and CO_2_ were determined using the analyzer. The activity of the samples was evaluated by measuring the percentage of CO conversion. The peak area of CO was examined before and after the test.

### 2.5. TOF-SIMS Test

Time-of-flight secondary ion mass spectrometry (TOF-SIMS) spectra and depth profiles of the palladium layers were acquired using a TOF-SIMS IV secondary ion mass spectrometer (IONTOF GmbH, Münster, Germany) equipped with a high mass resolution time-of-flight mass analyzer and Bi liquid metal ion gun. A high-current bunched mode of primary 25 keV Bi_3_^+^ ion beam operation was used during analysis. The primary ion beam pulse width was 1 ns, the repetition frequency was 10 kHz, and the average current was 0.2 pA. TOF-SIMS spectra were recorded for 30 s for a surface area of 50 × 50 µm^2^, giving an ion dose below the static limit of 10^13^ ions/cm^2^. For the depth profile analysis, a 300 × 300 µm^2^ area of the sample surface was sputtered using a 3 keV O_2_^+^ ion beam. The analyzed area of the sample surface was set to 50 × 50 µm^2^. The average current of the sputtering beam was 100 nA.

### 2.6. Characterization of the Catalyst with ICP

The samples were dipped in solutions with different concentrations of Pd. Wet chemical analysis was performed with inductively coupled plasma mass spectrometry (ICP-MS) using a Thermo Scientific X-Series (ICP-MS, UK). Due to the lack of the proper certificate reference material a second confirmatory technique based on the ICP plasma was used (Inductively Coupled Plasma Optical Emission Spectroscopy, ICP-OES by Thermo Scientific iCAP 7400, UK). Generally, there were no statistically significant differences in the Pd levels associated with the employment of various quantitative techniques based on plasma ICP. The Pd levels were too low for the ICP-OES technique to be measured precisely only in the case of the lowest PdCl_2_ concentration. Prior to ICP analysis, the material was treated with a mixture of acids in a microwave oven system (UltraWAVE by Milestone, Italy). Amounts of approximately 0.08 g of each sample were added to glass vials. The samples were mineralized in two replicates (each Ti wire coated with Pd was cut into two pieces). We used reverse aqua regia to decompose the surface layers. Subsequently, we added 3 mL of concentrated 65% nitric acid (BAKER ANALYZED, Ultra-Pure, USA) and 1 mL of concentrated hydrochloric acid (Chempur, Ultra-Pure, Poland). A blank test containing no sample was prepared in the same way.

After decomposition, the samples were quantitatively transferred to class “A” flasks. The flasks were filled with deionized water up to a final volume of 100 mL. The process of mineralization consisted of two steps:

- Stage I (20 min): maximum pressure inside the reactor 130 bar; maximum temperature inside the reactor 230 °C; maximum microwave power 1500 W. Final temperature 230 °C.
- Stage II (10 min): maximum pressure inside the reactor 130 bar; maximum temperature inside the reactor 230 °C; maximum microwave power 1500 W. Final temperature 230 °C. The sample holder was placed in a Teflon container filled with water. This ensured the same conditions and access to microwave energy. Before sample decomposition, the whole reactor was pumped with nitrogen gas under a pressure of 40 bar. The gas phase acted as a cap for the vials, preventing boiling and cross-contamination of the solutions.

For calibration of both instruments single element palladium standard solution by Peak Performance (USA) was used and subsequently diluted. 

Additionally, single element indium standard solution (by Merck, Germany) was used an internal standard and satisfactory recovery values were obtained (very close to 100%) for both ICP spectrometers. Along with the samples also blank sample was measured and no significant background increase connected with the use of the microwave oven system or chemical reagents was noticed. All measurements were carried out for a few isotopes of Pd (ICP-MS) and a few emission lines of Pd (ICP-OES). The final results were calculated against the ^105^Pb isotope (ICP-MS) and emission line of Pd at 340.458 nm in axial position of the torch (ICP-OES).

### 2.7. Characterization of the Catalyst with SEM

The structure of the porous layer was investigated with scanning electron microscopy (SEM). A scanning electron microscope (SEM S-4700, Hitachi, Tokyo, Japan) was used equipped with an energy dispersive spectrometer (EDS, Thermo-Noran Inc., Madison, WI, USA) or back-scattered electron with yttrium–aluminum garnet detector (YAG-BSE, Thermo-Noran Inc., Madison, WI, USA). To study the structure and composition of the surface, samples in the form of a wire were examined with two types of detectors, YAG-BSE and EDS.

### 2.8. Characterization of the Catalyst with XRD

A PANalytical X’Pert Pro MPD diffractometer in the Bragg–Brentano reflection geometry was used to study the X-ray diffraction patterns of the samples at room temperature. Copper CuKα radiation from a sealed tube was used. Data were collected in the 2θ range of 5–90° with a step of 0.0167° and exposure per step of 27 s. The samples were spun to minimize preferred orientation effects. A PANalytical X’Celerator detector based on real-time multiple strip technology was used. This detector is capable of simultaneously measuring intensities in the 2θ range of 2.122°. The PANalytical High Score Plus software package was used for qualitative analysis and to estimate the size of crystallite ions, in combination with the powder diffraction file (PDF-2 ver. 2009) from the International Centre for Diffraction Data (ICDD) database of standard reference materials.

## 3. Results and Discussion

Many factors can influence the activity and selectivity of metallic palladium catalysts. The kind of carrier used and how the catalysts are obtained may influence the catalytic properties of supported metallic systems.

**Figure 2:**
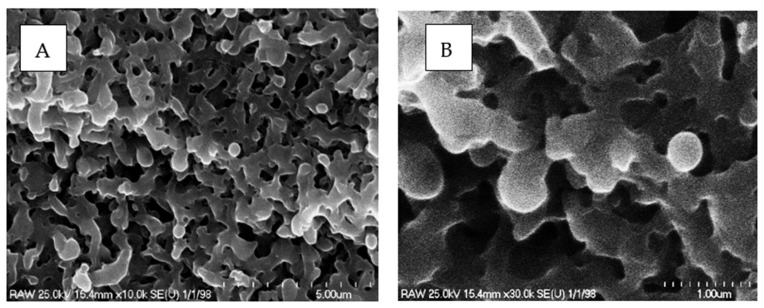
Scanning electron microscope (SEM) analysis of porous layer at different magnifications: (**A**) 10 K; (**B**) 30 K.

**Figure 3:**
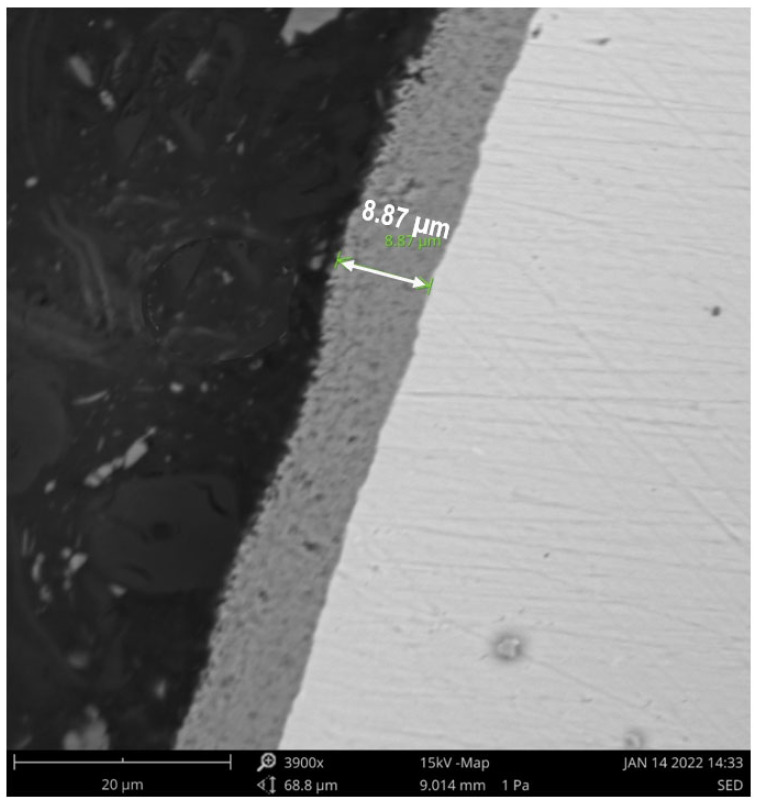
Scanning electron microscope (SEM) image of porous layer sample, cross-section view.

**Figure 4:**
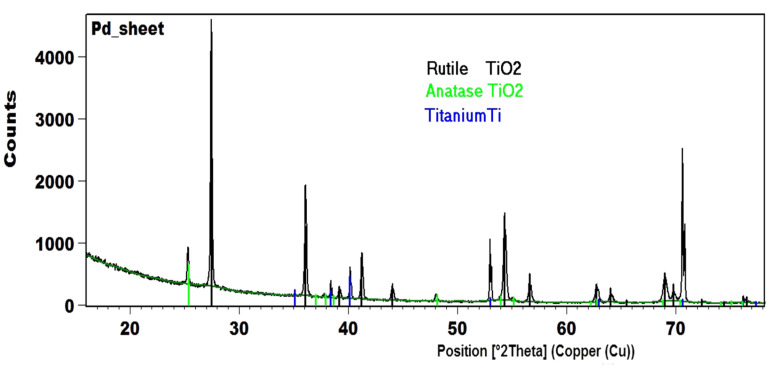
XRD patterns of TiO_2_/Ti after PEO processes: **−−** TiO_2_ rutile; **−−** TiO_2_ anatase, **−−** Ti.

In the first stage of our research, the PEO process was used to produce a TiO_2_ porous layer with a well-developed surface on a commercial titanium wire. The structure of the porous layer was studied with scanning electron microscopy (SEM). The results are shown in Figure 2. A cross-section view of the sample is presented in Figure 3. The TiO_2_ layer had a thickness of approximately 9 µm and was characterized by a regular distribution of pores. Based on Figure 2B, the diameter of the pores was typically less than 1 µm, which is desirable from a catalytic point of view, since such structures can readily transport reactants to the active sites. X-ray powder diffraction (XRD) tests were used to examine the phase composition of the obtained oxide layer and assess its suitability as a carrier for catalysts. The XRD patterns of samples of TiO_2_/Ti after PEO are presented in Figure 4. Rutile and anatase crystalline phases were identified in the surface layer of TiO_2_, which suggests this material is a promising support for metal catalysts.

In general, the properties of the metal-supported catalysts were closely related to the dispersion of the active metal. The most active catalysts were obtained when the active phase was in the form of very small crystallites of up to a few nanometers dispersed on the surface of a support. The impregnation method is commonly used for the preparation of Pd catalysts. This method requires that the support is first treated with a solution of the metal precursor, usually a salt, before being aged (usually for a short time), dried, and calcined. Depending on the amount of solution used, two types of impregnation can be distinguished: dry impregnation and wet impregnation. Regardless of the type of impregnation used, the Pd particles are usually large and dispersed nonuniformly, especially in the case of catalysts with high Pd loading [52,53].

**Figure 5:**
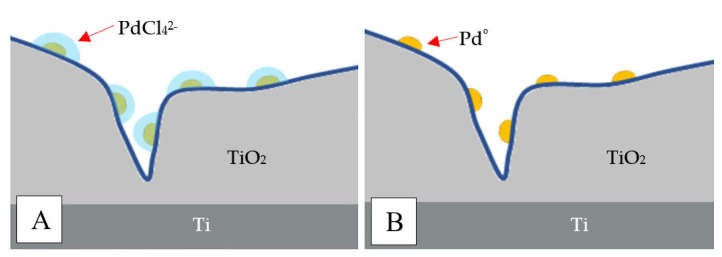
Effect of H_2_ reduction on Pd active sites: (**A**) before reduction; (**B**) after reduction.

An alternative method of preparing Pd catalysts on oxide supports is the adsorption method. This procedure is particularly useful when precursor–support interactions are anticipated. Inorganic oxides, including TiO_2_, tend to polarize and become-surface charged once they are suspended in an aqueous solution. The charge is determined by the pH. In acidic media, the adsorption surface site (M–OH) is positively charged and would be covered by anions. In basic media, the acidic surface site (M–OH) would be negatively charged and covered by cations. For each oxide, there would be a peculiar pH at which there is no surface charge. This pH is called the PZC (zero point of charge) or IEPS (isoelectric point) [54]. PZC values are available in the literature [55,56]. In the case of TiO_2_, the PZC point is at a pH of ~6 [56]. Palladium is known to form PdCl_4_^2−^ anions in acidified solution with HCl [57,58]. Therefore, we acidified the solution of PdCl_2_ with HCl up to pH 4 (excess Cl^-^ ions were necessary for the formation of PdCl_4_^2−^ ions) and performed adsorption on the centers of Ti^n+^ on the TiO_2_ phase in different concentrations of Pd% (0.02–2% wt.) (Figure 5A). To obtain Pd/TiO_2_/Ti catalysts, the precursors were reduced in H_2_ at 400 °C for 2 h (Figure 5B).

In the adsorption method, a porous material (a powder, a construction material covered with a layer of porous material) is immersed in a metal solution and left for a specified time. When the sample is removed from the solution, it is washed with a solvent and dried. The quantity of metal ions remaining on the porous material is determined by the number of active centers on the surface of the support. Since not all areas of the surface are energetically equivalent, there are groups of energetically privileged atoms on which the adsorption of palladium ions occurs. Therefore, it is necessary to determine the quantitative composition of the final catalyst. The ICP-MS technique was used to determine the Pd content of the Pd/TiO_2_/Ti catalysts. The results are summarized in Table 1.

**Figure 6:**
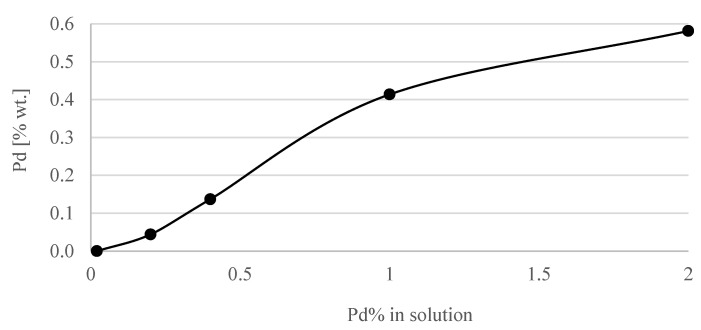
Adsorption of palladium on the surface of the TiO_2_/Ti support.

Figure 6 shows the dependence of the amount of adsorbed Pd on TiO_2_/Ti as a function of the concentration of PdCl_4_^2−^ ions in the solution. The relationship was not linear. As the concentration of Pd ions in the solution increased, the amount of palladium atoms adsorbed on the surface increased. However, the increases became smaller. It follows that there would be a concentration of ions beyond which there would be no further increase in the amount of metal in the catalyst. All adsorption centers would be saturated.

On the other hand, it can be assumed that the increase in the concentration of ions in the solution would cause the accumulation of further metal layers on the surface, and, consequently, an increase in palladium particles. Such a state would increase the amount of palladium in the digested and measured samples of Pd/TiO_2_/Ti using the ICP-MS technique, but would not increase the activity of the catalysts. The mass of Pd in the system would increase, but the number of Pd centers available on the surface would not increase. To explain this phenomenon, we tested the catalytic activity of the Pd/TiO_2_/Ti systems in the CO oxidation reaction.

**Figure 7:**
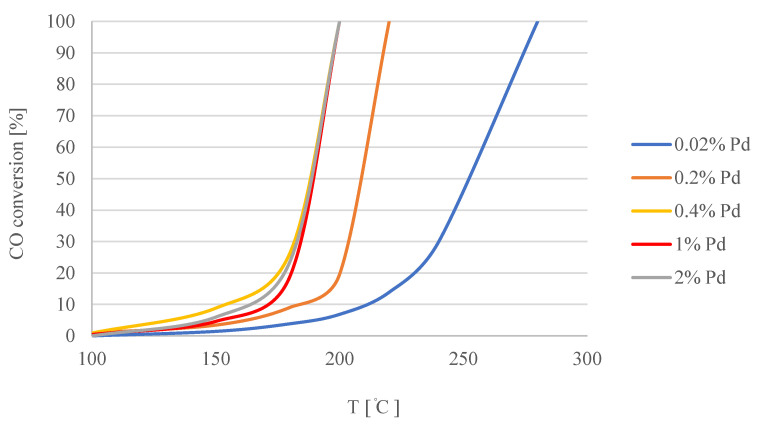
Catalytic activity of samples of Pd/TiO_2_/Ti catalysts with different amounts of palladium.

The activity of the catalysts was tested in the presence of a mixture of synthetic air and 5% CO. The tests were continued until no CO was present in the outlet gas from the GC-TCD. The results are presented in Figure 7. In the temperature range from room temperature to 450 °C, the CO conversion on the Ti metal supports with a PEO layer only (without Pd particles) was no more than 5%. The Pd/TiO_2_/Ti catalyst samples prepared in a solution with 0.4% Pd were examined ten times. During each cycle, the temperature was increased up to 200 °C. The test was conducted for 4 h, and 100% conversion was achieved in each cycle.

**Figure 8:**
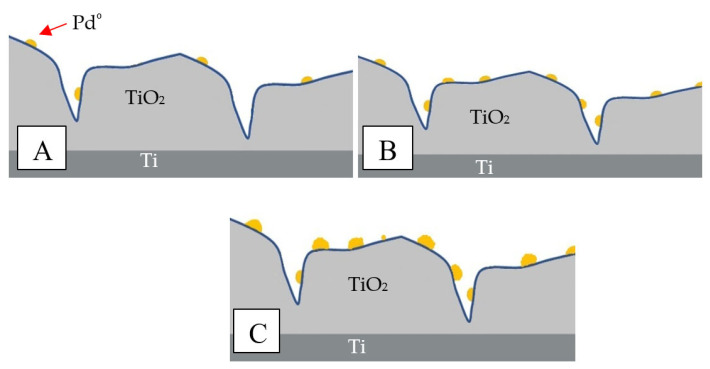
Comparison of catalytically active sites with different amounts of Pd: (**A**) 0.02% wt., (**B**) 0.2% wt., (**C**) 2% wt.

The adsorption of CO on Pd is known to be quite strong. Therefore, the conversion of CO to CO_2_ at lower temperatures (<100 °C) is slow, and higher temperatures are needed in order to activate the catalyst [59,60,61]. By increasing the concentration of Pd in the solution up to 0.4%, the activity of the samples was increased (Figure 7). Increasing the amount of Pd above 0.4% did not change the activity of the samples. This could be the result of the agglomeration of Pd on the porous surface. In Figure 8, Pd active sites on the Ti/TiO_2_ support are presented as yellow dots. When the concentration of Pd ions in the solution increased from 0.02% wt. to 0.2% wt., the adsorption centers on the TiO_2_ surface became saturated (Figure 8B). As a result of the reduction of precursors in H_2_ at 400 °C, more small-sized palladium crystallites were formed on the surface of the Pd/TiO_2_/Ti catalysts, increasing their activity. Further increasing the concentration of PdCl_2_ in the solution during adsorption caused the deposition of palladium ions on the same centers. As a result of the reduction of precursors, we obtained the same amount of larger Pd particles on the surface (Figure 8C), which caused only a slight increase in the amount of surface Pd atoms involved in the oxidation of CO. No further increase in the CO activity of such systems was visible.

**Figure 9:**
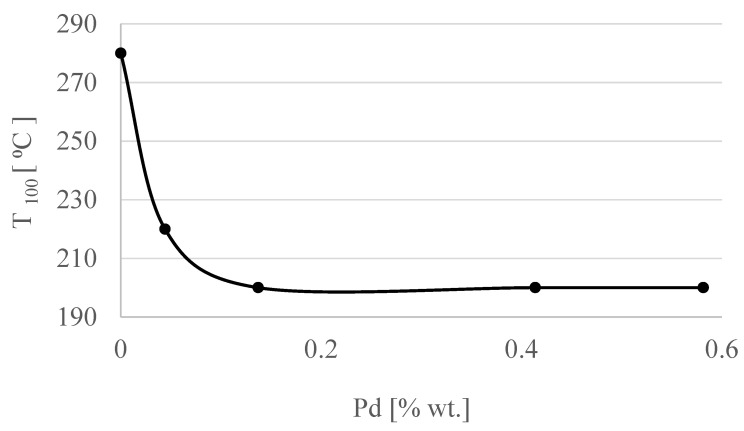
Relationship between T_100_ (°C) and Pd (% wt.).

The relationship between the amount of Pd and T_100_ (°C) is presented in Figure 9. It can be concluded that increasing the amount of Pd up to 0.137 (% wt.) increased the activity, but adding more Pd did not result in further improvements.

**Figure 10:**
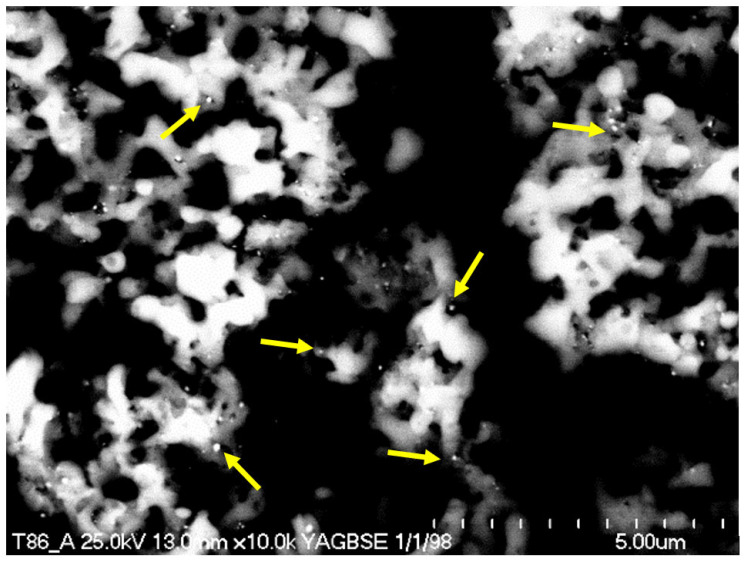
Backscattered electron–scanning electron microscope (BSE-SEM) image of Pd/TiO_2_/Ti catalyst prepared with adsorption of Pd ions from a solution with 2% Pd-wt. The yellow arrows indicate Pd particles on the surface.

To determine the distribution of palladium particles on the surface of the catalysts, SEM tests were performed with BSE and EDS detectors. Using the SEM-BSE analysis, the amount of Pd on the samples prepared in solutions with less than 1% Pd was below the level of detection. The presence of Pd crystallites was only detected in the case of the sample prepared in a solution containing 2% Pd wt. (Figure 10). In the BSE-SEM image, a variety of Pd particles (indicated by yellow arrows in Figure 10) was observed on the surface of the TiO_2_/Ti support. This confirmed the uniform distribution of Pd on the surface. In other words, adsorption led to palladium systems with uniform distributions of large Pd particles on the surface.

**Figure 11:**
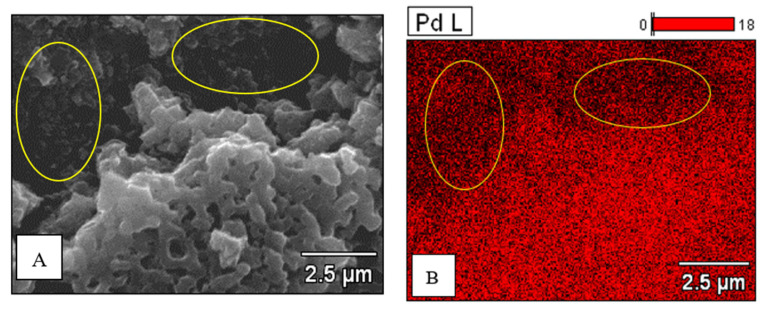

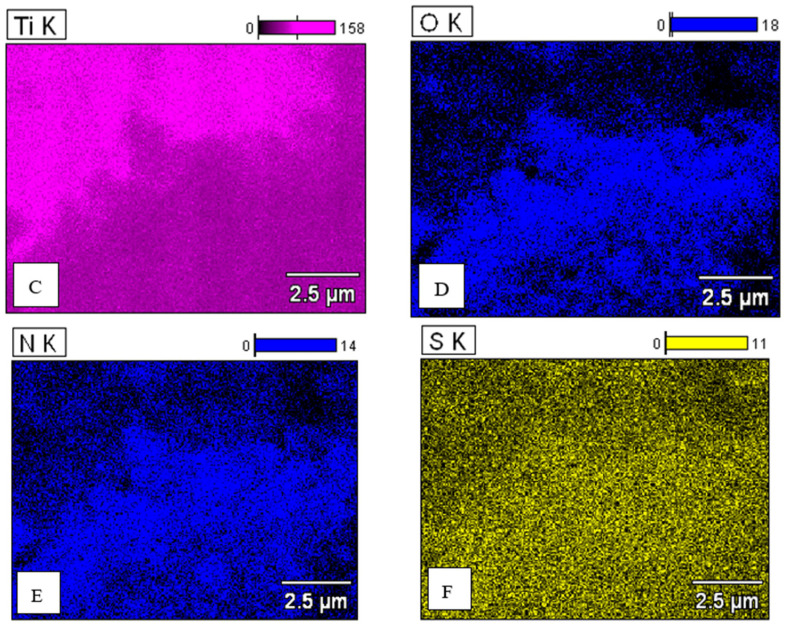
Scanning electron microscope–energy dispersive spectrometry (SEM-EDS) images of Pd/TiO_2_/Ti catalyst obtained with adsorption of Pd ions from 2% Pd wt. solution. (**A**) images; (**B**–**F**) elemental analysis.

The adsorption of Pd may occur only on the TiO_2_ surface. Figure 11 shows an elemental map of Pd in two areas where a part of the porous layer was intentionally removed. In the area indicated with oval markers in Figure 11A, the porous layer was peeled off. As can be seen in Figure 11B, the intensity of the Pd map was noticeably lower. As shown in Figure 11C,D, respectively, the maps of titanium and oxygen in the same area were correlated with the presence of oxygen on the surface of the samples. This was confirmation that titanium oxide formed during PEO due to the combination of Ti and O_2_.

**Figure 12:**
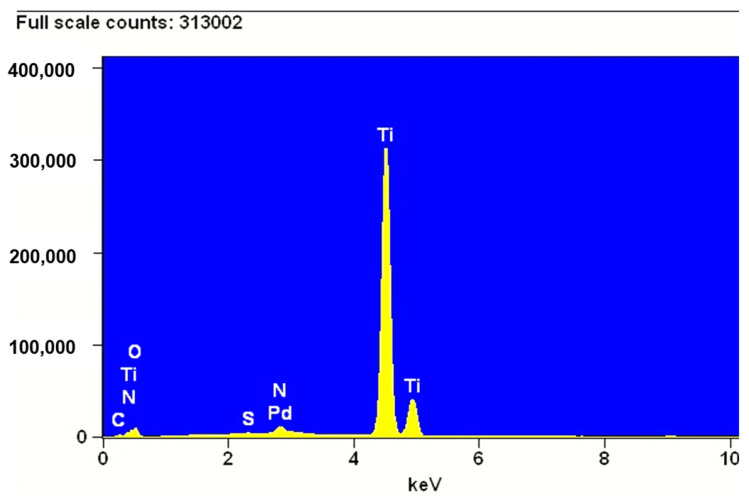
Energy dispersive spectrum of Pd/TiO_2_/Ti catalyst obtained with adsorption of Pd ions from 2% Pd wt. solution.

Figure 12 shows the EDS spectrum of the Pd/TiO_2_/Ti catalyst obtained with adsorption of Pd ions on a titanium wire covered with a layer of TiO_2_. The spectrum shows a maximum that can be attributed to Pd, which confirms the presence of this metal on the surface of the systems. This result could not be confirmed with the XRD method, which is not suitable for testing catalysts containing less than 1 wt.%. Pd.

**Figure 13:**
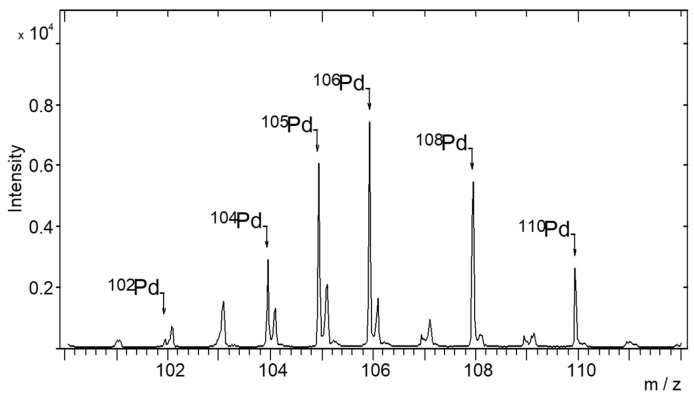
TOF-SIMS spectrum of palladium isotope ions emitted from the surface of Pd/TiO_2_/Ti catalyst obtained with adsorption of Pd ions from 2% Pd wt. solution.

The time-of-flight secondary ion mass spectrometry (TOF-SIMS) technique was used to study the distribution of Pd particles in more detail. Figure 13 presents an exemplary TOF-SIMS spectrum of a Pd/TiO_2_/Ti sample for an m/z range corresponding to Pd isotopes.

**Figure 14:**
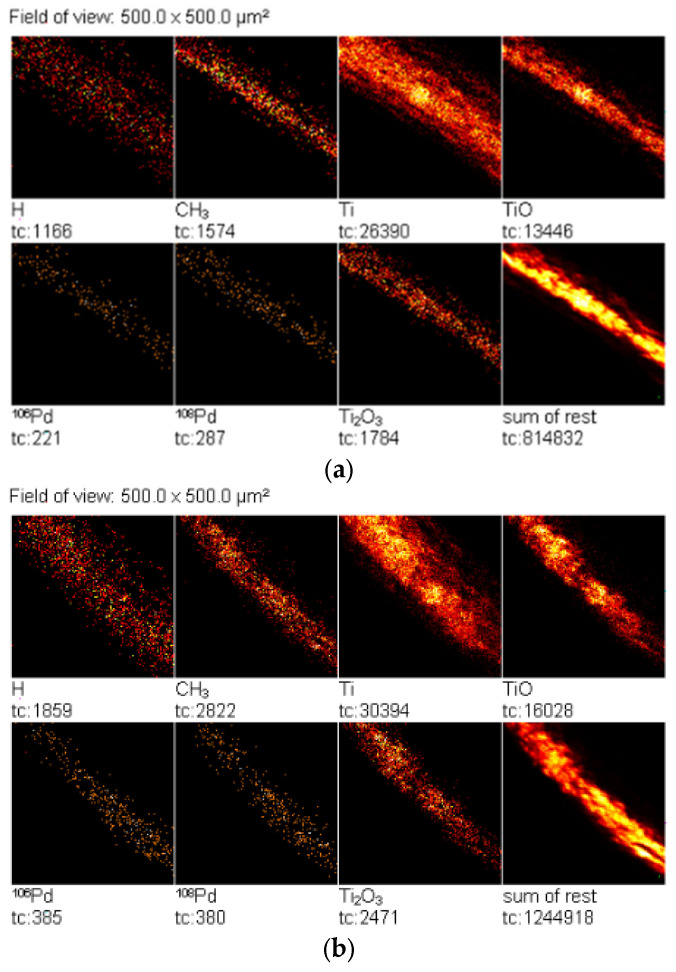

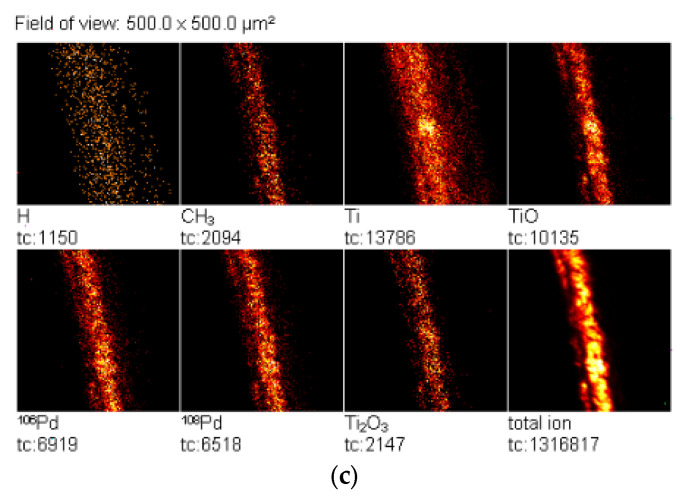
TOF-SIMS images of the surfaces of Pd/TiO_2_ catalysts obtained from solutions containing (**a**) 0.02, (**b**) 0.2, and (**c**) 2% of Pd. The ion count (tc) of secondary ions is shown under each image.

The TOF-SIMS images in Figure 14 show that the Pd was uniformly distributed on the surface of the Pd/TiO_2_/Ti catalyst obtained from solutions with different Pd concentrations. At the same time, it can be concluded that the amount of palladium ions removed from the surface of the catalysts increased with higher concentrations of Pd^2+^ ions in the solutions.

**Figure 15:**
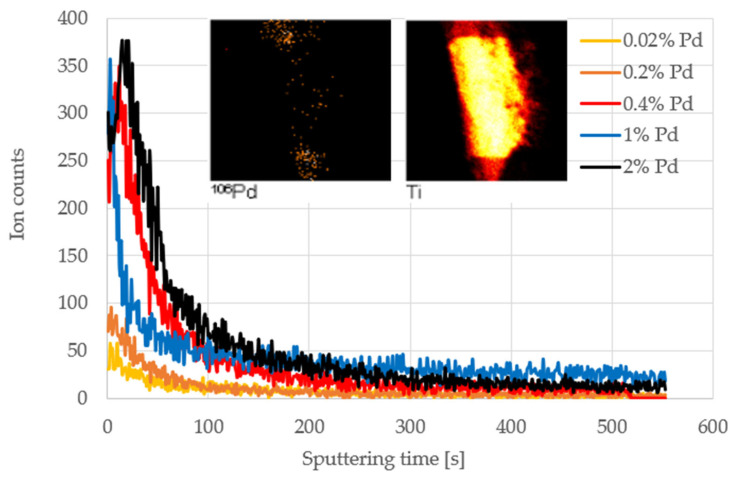
TOF-SIMS depth profiles of ^106^Pd secondary ion emission from samples obtained from solutions with different Pd concentrations. Inset: TOF-SIMS images of Pd^+^ and Ti^+^ ions recorded after completion of depth profile acquisition for the Pd/TiO_2_ catalyst obtained from the 0.02% solution.

TOF-SIMS depth profiling was used to study the distribution of Pd in the near-surface region. The results are presented in Figure 15. As can be seen, Pd-enriched layers were successfully formed on the surface of all the catalysts. The Pd content in the surface layers increased with the concentration of Pd in the solution. This trend was visible up to the level of 0.4% Pd wt. in the solution. Increasing the concentration of palladium in the solution resulted in a wider palladium layer. The width of the palladium layer was estimated in terms of the sputtering time at which Pd^+^ emission decreased to half of its initial value, recorded at the beginning of the depth profile. This method of approximation was necessary due to the shape of the catalyst.

TOF-SIMS was also used to obtain information concerning palladium dispersion on the surface of the Pd/TiO_2_ catalysts. It was assumed that the total area of the Pd–TiO_2_ interface of catalysts with a higher dispersion (characterized by a lower average Pd particle size) would be larger than in the case of the catalyst with a lower dispersion (containing larger particles on average). Relative values of the emission intensity of PdTiO_2_^+^ ions characteristic for the composition of the Pd–TiO_2_ interface to the emission intensity of Pd^+^ ions were chosen as a measure of the area of this region of the catalyst. TOF-SIMS emission intensities of PdTiO_2_^+^ and Pd^+^ ions for different Pd/TiO_2_ catalysts are presented in Table 2.

**Figure 16:**
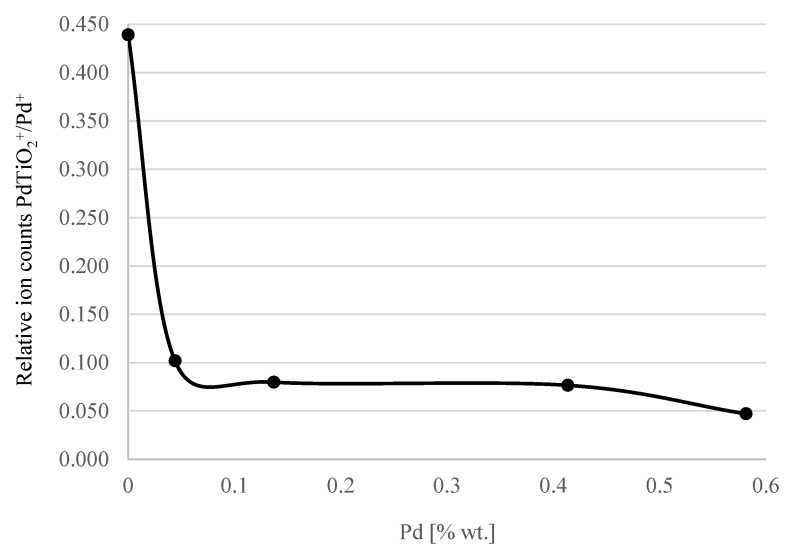
PdTiO_2_^+^ to Pd^+^ ion intensity ratio as a function of Pd content in the catalyst.

The PdTiO_2_^+^ to Pd^+^ ion intensity ratio as a function of Pd content presented in Figure 16 corresponded to the changes in the Pd/TiO_2_/Ti catalyst activity as a function of the Pd content in the solution presented earlier in this article (Figure 9). This suggested that the observed changes in the catalyst activity depended on Pd dispersion, which in turn was controlled by the concentration of Pd in the solution.

In summary, the proposed Pd/TiO_2_/Ti catalysts could be easily formed into any shape, and the obtained porous layer with applied Pd was durable. The catalysts showed activity in the process of CO oxidation at a relatively low temperature. The systems were low-loaded with Pd and a 100% CO conversion was achieved at the lowest temperature by a catalyst containing only approximately 0.14% Pd wt. The catalyst was also stable, as confirmed by activity tests performed ten times, during which no decrease in activity was noted. These results clearly indicated the potential of using Pd/TiO_2_/Ti catalysts in industrial applications.

## 4. Conclusions

In this study, PEO and adsorption methods were applied to produce Pd/TiO_2_/Ti catalysts. The PEO technique was used to create a uniform porous TiO_2_ layer on a Ti construction material (Ti commercial wire). Palladium was applied to the porous structure using adsorption techniques. The optimum conditions for Pd adsorption were achieved using a solution with 0.4% of PdCl_2_ acidified to pH ~4 with the addition of HCl. After the reduction of this catalyst in H_2_ at 400 °C for 2 h, the content of Pd was determined with ICP-MS at nearly 0.14% Pd wt. The catalyst reached a 100% conversion of CO at 200 °C and was stable under the reaction conditions. The proposed method of preparing active palladium catalysts on titanium construction materials (pipes and sheets) shows potential for industrial applications.

## Figures and Tables

**Table 1:** Amount of Pd in Pd/TiO_2_/Ti catalysts.

Concentration of Pd in Solutions (%)	Amount of Pd in Catalyst (% wt.)
0.02	<0.001
0.2	0.044
0.4	0.137
1	0.414
2	0.581

**Table 2:** Secondary ion counts for Pd/TiO_2_ catalysts.

% Pd in Solution	TOF-SIMS Secondary Ion Counts		Relative Values of Ion Counts
	Pd^+^	PdTiO_2_^+^	PdTiO_2_^+^/Pd^+^
0.02	565	248	0.439
0.2	2621	267	0.102
0.4	8400	670	0.08
1	3917	300	0.077
2	12276	578	0.047

## Data Availability

Not applicable.
